# The Biomechanics of Spinal Orthoses for Adolescent Idiopathic Scoliosis: A Systematic Review of the Controlling Forces

**DOI:** 10.3390/bioengineering11121242

**Published:** 2024-12-08

**Authors:** Changliang Luo, Huidong Wu, Wei Liu, Yuyan Luo, Yi Jie, Christina Zong-Hao Ma, Mansang Wong

**Affiliations:** 1 Department of Prosthetic and Orthotic Engineering, School of Rehabilitation, Kunming Medical University, Kunming 650032, China; 22039265r@connect.polyu.hk (C.L.); huidong_wu@126.com (H.W.); weiscu.liu@connect.polyu.hk (W.L.); 2 Department of Biomedical Engineering, The Hong Kong Polytechnic University, Hong Kong SAR 999077, China; yuyan-laura.luo@connect.polyu.hk (Y.L.); yi620.jie@connect.polyu.hk (Y.J.); czh.ma@polyu.edu.hk (C.Z.-H.M.); 3 Research Institute for Smart Ageing, The Hong Kong Polytechnic University, Hong Kong SAR 999077, China

**Keywords:** AIS, biomechanics, corrective force, spinal orthosis

## Abstract

Background: Orthotic treatment is a well-acknowledged conservative treatment for moderate adolescent idiopathic scoliosis (AIS). The efficacy of this treatment is significantly determined by the forces applied to the bodies of patients. However, there is uncertainty regarding the optimal force levels that should be applied to the patient’s torso by spinal orthosis. This study aims to identify reference values for the controlling forces in AIS management. Methods: A comprehensive literature search was performed in five databases (PubMed, Scopus, Cochrane Library, CINAHL, and Web of Science). Only studies written in English and covering the force/pressure measurements of spinal orthosis for the treatment of AIS were included, without publication date restrictions. The methodological index for non-randomized studies (MINORS) was employed for the methodological quality assessment, and force measurements were standardized to pressure in kilopascals (kPa) for comparison. Results: From the initial 10,452 records, 10 studies were admitted for the final analysis. All the included studies reported the interface pressure between the thoracic (T) pad and patient’s trunk, and seven studies evaluated the pressure from the thoracolumbar/lumbar (TL/L) pad. These studies used different pressure sensors or transducers with the range from 5.6 to 82.5 kPa for the T pads, and 4.8 to 85.1 kPa for the TL/L pads. Four studies reported strap tensions of 26.8 to 60.4 N. Higher strap tension was correlated with increased interface pressure (r = 0.84). Conclusion: The mean strap tension was 42.5 N, the median interface pressure of the T pads was 8.75 kPa, and the median pressure of TL/L regions was 7.11 kPa without the outliers. The findings provide a baseline value for designing adjustable straps and strategically distributing pressure in orthoses.

## 1. Introduction

Adolescent idiopathic scoliosis (AIS) is a common three-dimensional (3D) spinal deformity that is usually managed with spinal orthosis for moderate cases [1,2]. Orthotic treatment has been proven to be effective in stopping the curve progression before skeletal maturity [3]. Notably, the fundamental mechanical principle for the orthotic treatment of AIS is mainly about the application of the controlling force. The goal of the correction is to reshape the scoliotic spine to its natural alignment while preventing damage to the nervous system. This could be achieved by applying the proper controlling forces to the spine with the use of orthoses. The forces needed to correct the deformity must be sufficient to achieve the optimal correction [4]. By exerting force on specific points of the trunk, the orthotic treatment endeavors to induce mechanical modifications to the shape of the spine and control the curve progression [5].

Various types of spinal orthoses have been developed with different designs and correction mechanisms, such as Milwaukee orthosis [6], Boston orthosis [7], and Cheneau orthosis [8]. The efficiency of these orthoses varies depending on the different designs [9]; the characteristics of patients [10,11]; the severity, location, and patterns of the curves [12,13,14]; and other influential factors (e.g., compliance [3,15] and spinal flexibility [16]). The orthoses designs are primarily based on the use of endpoint control, transverse forces, traction forces, and combinations of both lateral and vertical forces [17].

The measurement of controlling forces in spinal orthosis is a key aspect of curve progression control and corrective effect prognosis for patients with AIS. The quantification of the interface force may help to design optimum pads and orthosis, establishing some links with corrective outcomes. However, the complexity of the human body environment hinders the assessment of the forces [18,19]. Still, some progress was made in the past decades on how forces were given, how forces were measured, and how much force was applied.

Some researchers measured the contact pressure at the orthosis–trunk interface [20,21], and some measured the tensions in the straps [22]. Aubin et al. proposed a correction scheme consisting of the application of forces to the anterior rib hump and on the convex side at the thoracic apical level while preventing the posterior displacement of the rib hump [23]. Karami et al. found that the combination of vertical and transverse directed forces was effective in decreasing the scoliotic curve without influencing the curves in the sagittal plane [12]. A pressure distribution measurement system was utilized in Romano’s study to detect pressure between the pad of the orthosis and the rib hump [24]. Chung et al. used a robotic testing platform with six-axis load cells to quantify the corrective force in Boston orthosis [25]. Some insole pressure testing devices were also applied to the trunk–orthosis interface pressure measurements, such as the TekScan system [26] and Pedar system [27]. In addition, the force or pressure magnitude for providing a correction effect has also been investigated in several studies. Pham et al. measured the pressure values in the standing position before and after strap tightening: for the thoracic curve, the pressures were 7.99 kPa and 8.8 kPa, while for the thoracolumbar curves, the values were 7.04 kPa and 7.65 kPa [26]. Clin et al. tested the strap tension from 20 N to 60 N, which increased the average correction of the thoracic and lumbar curves, and the maximal pressures exerted to the body were in the range of 0–35 kPa [28].

Although the effect of orthotic treatment has been extensively investigated and confirmed, the application of controlling forces is mainly dependent on the subjective experience of the orthotist, and there are no clear recommendations or guidelines regarding this. So, the aim of this literature review was to identify the reference values of the application of controlling forces to contribute to a more informed decision on manufacturing spinal orthosis. The findings will lead to a better understanding of the biomechanics of spinal orthotics, and guide both orthotists and researchers to be more precise when designing spinal orthoses or identifying proper measurements to quantify controlling forces for patients with AIS.

## 2. Materials and Methods

### 2.1. Search Strategy

This review is conducted in accordance with the guidelines of the Preferred Reporting Items for Systematic Reviews and Meta Analyses (PRISMA) [29]. The project protocol was registered in the International Prospective Register of Systematic Reviews (PROSPERO) with the registration number CRD42024613367. A comprehensive literature search was performed in the following five electronic databases on and before 2 August 2024: PubMed, Scopus, Cochrane Library, CINAHL, and Web of Science. The search strategy comprised three domains, with each domain encompassing multiple keywords that spanned the respective field. The strategy was limited to the English language with no publication date restriction. Gray literature was not included in this search. The following domains were used to identify the studies that investigated the biomechanics of spinal orthotics in AIS: (1) adolescent idiopathic scoliosis (AIS), idiopathic scoliosis (IS), scoliosis, spinal deformity, spinal disease, and spinal curvature; (2) orthotic (orthosis, orthoses, brace, and bracing); (3) biomechanics (force, tension, and pressure).

### 2.2. Study Selection and Eligibility Criteria

The study screening was based on the following criteria: (1) human subjects with AIS; (2) study with more than 5 cases; (3) subjects were treated with spinal orthosis, with quantified force/pressure applied; (4) full text was available. Any editorial, comment, letter, guideline, or protocol were excluded. The reference lists of the selected studies were manually searched to identify any other potential studies. Two independent reviewers searched and screened the records. Studies with the same author group were double-checked to ensure there were no duplicates.

### 2.3. Methodological Quality Assessment

The methodological index for non-randomized studies (MINORS) [30,31] was utilized to assess the methodological quality of all the included studies. For comparative research, the highest score is 24, while for non-comparative studies, it is 16. In studies that were not comparative, a score of 0–4 indicated extremely low quality, a score of 5–7 indicated low quality, a score of 8–12 indicated fair quality, and a score of ≥13 indicated high quality. In comparison studies, a score of 0–6 denoted extremely low quality, a score of 7–10 denoted low quality, a score of 11–15 denoted fair quality, and a score of ≥ 16 denoted high quality.

Quality assessment of the literature was carried out by two independent reviewers, and in case of uncertainty about the results, the decision was discussed with a third reviewer.

### 2.4. Data Collection and Analysis

Data were extracted and categorized from the included studies based on the following elements: (1) study identification, including first author, year of publication, and country; (2) study settings; (3) subject characteristics, including sample size, gender, mean age, Cobb degrees, and curve type; (4) biomechanics information, including force/pressure type, force/pressure magnitude, and force/pressure measurement method; (5) type of orthoses; (6) study findings. If numerical results were available, these were given priority. In cases where numerical data were incomplete or unavailable, data were extracted from graphs. To be consistent, all the reported force magnitudes were converted to pressure in kilopascals (kPa) based on the force area.

Prior to the data collection, a customized form was subjected to a pilot test using three articles. This allowed for the identification of any potential missing data from the form, which was then adjusted and modified accordingly.

## 3. Results

### 3.1. Study Selection

**Figure 1:**
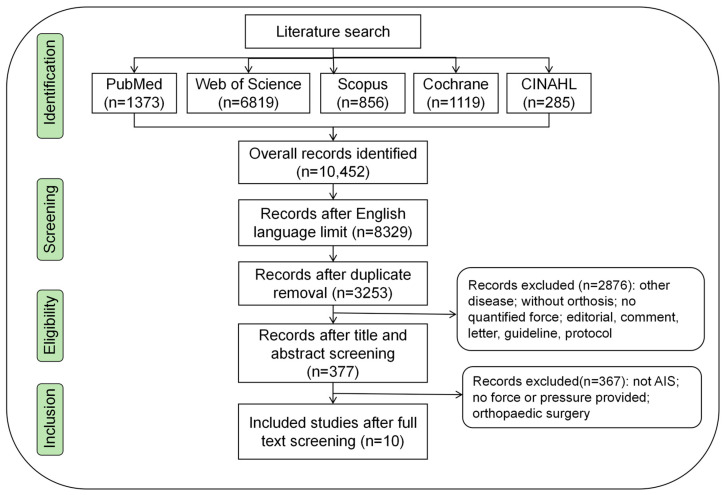
PRISMA flowchart.

A total of 10,452 articles were retrieved from the databases after English filtering and duplicate removal. A total of 10,075 articles were excluded based on the eligibility criteria. After conducting the full-text screening of the remaining 377 articles, 10 articles were included in the final analysis. The study selection process and the reasons for exclusion are presented in Figure 1.

### 3.2. Basic Characteristics of Included Studies

Among the ten included studies, all the articles reported the interface pressure (IP) between the thoracic (T) region of spinal orthosis and the trunk [7,20,26,27,32,33,34,35,36,37], six studies reported the force between the thoracolumbar/lumbar (TL/L) pads and the trunk [7,20,26,27,34,36], and four studies measured the strap tensions (ST) [7,32,33,35]. The total sample size included in this review is 292, ranging from 8 to 72 per study. Overall, eight countries or regions reported relevant results, including 44 subjects from Greece, 32 subjects from France, 35 subjects from Hong Kong, 30 from the USA, 49 were from Canada, 72 were from Malaysia, 14 were from England, and 16 of the subjects were from the Netherlands. In addition, eight studies were conducted two decades ago [7,20,26,27,32,33,35,37], one was published in 2011 [36], and one was in 2019 [34]. Meanwhile, three studies were in laboratory experimental settings [26,32,33], and seven studies were conducted in clinical situations [7,20,27,34,35,36,37]. The basic characteristics of the 10 studies are summarized in Table 1.

### 3.3. Quality and Bias Assessment of Included Studies

Two reviewers assessed the quality of the included studies independently by using the MINORS criteria. All the included studies are non-comparative studies; three studies had scores of 8–12, indicating fair quality, and the rest seven studies had MINORS scores ≥ 13, suggesting high quality (shown in Table 2).

### 3.4. Force/Pressure Magnitudes

#### 3.4.1. The Strap Tension

**Figure 2:**
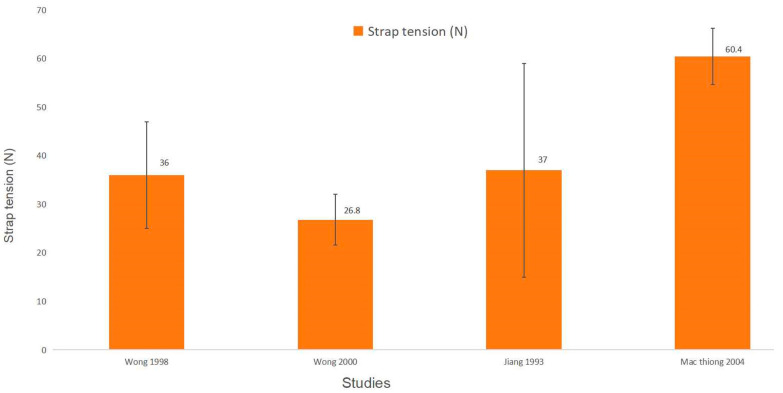
The strap tension of the included studies.

The tightness of the straps of a spinal orthosis is typically adjusted to achieve the desired level of support and stabilization for the orthosis while affecting the corrective forces exerted on the spine. The strap tension has been studied in four articles. Detailed information about the strength of strap tension is presented in Table 3 and Figure 2.

Wong et al. set three levels of thoracic strap tensions at 19 N, 38 N, and 49 N to investigate the effect of different strap tensions on the interface force from the thoracic pad; they measured a mean strap tension magnitude of 36 N, and found that when the tension of straps increased, the resultant force applied to the body by the thoracic pad increased as well [32]. Meanwhile, their other study reported the mean strap tensions of 26.8 N, and suggested that the tightness with which the orthosis was secured played a crucial role in influencing the biomechanical function of spinal orthosis [33].

Different magnitudes of strap tension have been evaluated, the approximate range could be identified as 26.8 N to 60.4 N based on the current literature [7,32,33,35], with a mean strap tension of 42.5 N.

#### 3.4.2. Thoracic Pad Pressure

**Figure 3:**
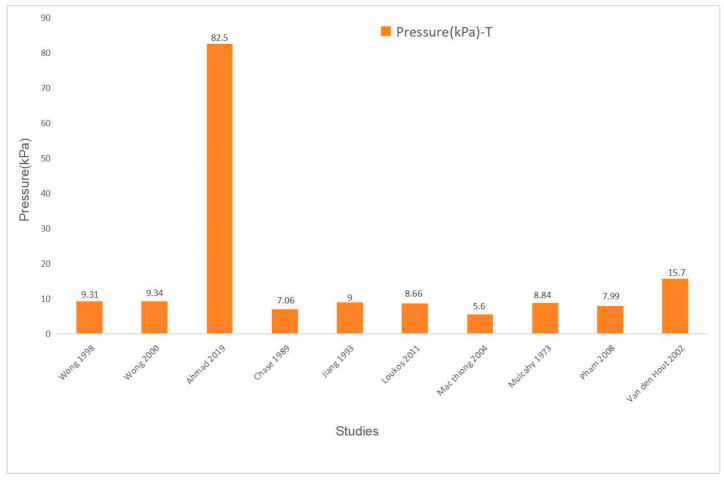
The pressure of the T pads of the included studies.

All ten studies reported the pressure applied by spinal orthosis pad on patients’ bodies. The pressure was primarily measured under the thoracic pad. Four studies measured the pressure of the Boston orthosis, two studies measured Cheneau orthosis, two others were Milwaukee orthosis, one was Hong Kong orthosis, and the remaining one had no clear description of the orthosis type. A maximal pressure magnitude was found in Ahmad’s study with the value of 82.5 kPa, which was exerted to the right thoracic at the task of the maximum strap tension [34]. Seven studies reported pressures within a range of 7 kPa to 10 kPa [20,26,32,33,35,36,37]. All the included studies detected the mean pressure magnitude value of the thoracic pads, ranging from 5.6 kPa to 82.5 kPa, with a median value of 8.92 kPa (inter-quartile range (IQR): 7.99 to 9.34). When excluding the outlier of 15.7 kPa [27] and 82.5 kPa [34], the median thoracic pad pressure was 8.75 kPa (IQR: 7.53–9.16). The summary of the thoracic pad pressure is shown in Table 4 and Figure 3.

#### 3.4.3. Thoracolumbar/Lumbar Pad Pressure

**Figure 4:**
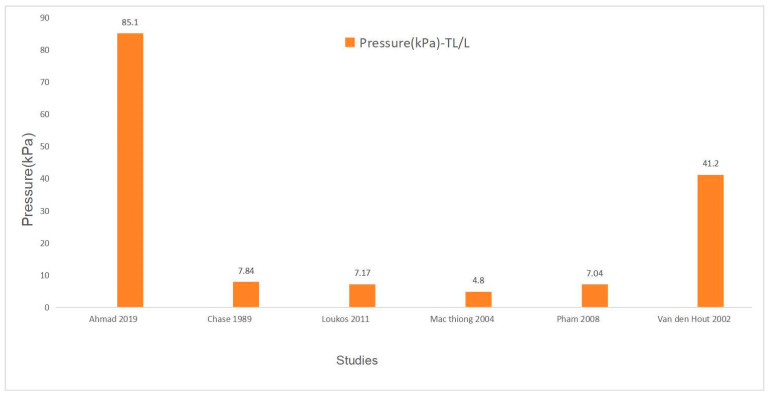
The pressure of the TL/L pads of the included studies.

Six studies reported the mean thoracolumbar/lumbar (TL/L) pad pressure of spinal orthoses [7,20,26,27,34,36]. Ahmad et al. identified the maximal pressure of 85.1 kPa [34]. Mac-Thiong et al. measured the minimal pressure of 4.8 kPa at the lumbar region [7]. The median value was 7.51 kPa, with an IQR of 7.04 to 41.2 kPa. After excluding the outliers from Van den Hout et al. and Ahmad et al.’s study [27,34], the median TL/L pad pressure was identified as 7.11 kPa (IQR: 5.92–7.51). The TL/L pressure magnitudes of each study are listed in Table 4 and Figure 4.

#### 3.4.4. The Pad Pressure and Strap Tensions of Different Orthosis Types, Age Groups, and Curve Patterns

Since the pressures extracted from Ahmad et al. [34] and Van den Hout et al.’s studies [27] were much greater than the other studies, the analyses of pad pressure and strap tensions of different types of orthosis, age groups, and curve patterns were conducted both with and without the inclusion of the outlier data from the two publications to address the influence of the extreme values (shows in Table 5).

Of all the included studies, five types of orthoses were used. The T pad pressure of Milwaukee orthosis, Boston orthosis, Chêneau orthosis, Hong Kong orthosis, and DDB orthosis were 9.08 kPa, 7.22 kPa, 7.99 kPa, 9.34 kPa, and 8.66 kPa, respectively, without the inclusion of outliers. The TL/L pad pressure of Boston orthosis, Chêneau orthosis, and DDB orthosis were 6.32 kPa, 7.04 kPa, and 7.17 kPa, respectively. Meanwhile, the strap tensions of Milwaukee orthosis, Boston orthosis, and Hong Kong orthosis were identified with 36 N, 48.7 N, and 26.8 N.

The ages of the patients from the included studies were categorized into four groups. In only one study with a mean age of 11 to 12 [34], the T pad pressure was 82.5 kPa and the TL/L pad pressure was 85.1 kPa. Three studies [26,35,36] reported the pressure magnitudes of the patients with a single curve and three other studies [27,32,34] measured the pressure of patients with double curves. For the single curve without the outliers, the T and TL/L pad pressures were 8.55 kPa and 7.11 kPa, and the strap tension was 37 N, while the T pad pressure, TL/L pad pressure, and strap tension were 7.06 kPa, 7.84 kPa, and 36N for the double curves, respectively.

## 4. Discussion

This systematic review aimed to explore the current evidence regarding the controlling force or pressure to obtain a reference value of spinal orthosis in the management of AIS. Ten publications were included in the extensive review of the current study. Even though great disparities existed among the published data, this review first synthesized the pressure magnitudes of spinal orthosis for patients with AIS. The findings regarding strap tensions, pad pressures, and the corrective outcomes of spinal orthoses are discussed below.

### 4.1. Strap Tension

The approximate range of the strap tensions was identified as 26.8 N to 60.4 N. Most of the included studies found that the tightening of the straps produced an increase in the pad pressure [7,26,32,33,34,36]. Wong et al. placed buckle force transducers on the straps to record the tensile force, they found that the thoracic strap tension was significantly correlated with the interface pressure in different postures and activities [32]. Average strap tensions were evaluated to be 36N [32] and 26.8 N [33]. However, as straps in different regions cause different effects, a standard value of strap tension should be set for each strap and should be adjusted over time. Moreover, the findings of Petit et al. [38] were in line with those of Chase et al. [20], which showed that the variation in strap tensions would affect the forces applied by the pressure pads and even change the progression of scoliotic curves. Aubin et al. conducted a study to measure the strap tension with patients wearing their orthoses and to document the variations in strap tension in different positions [22]. The straps were instrumented with load cells to record the tension variability while patients performed nine positions corresponding to normal daily tasks. The results of their study indicated that the tensions of pelvic straps and thoracic straps were quite different, and the strap tension decreased significantly in most activities compared to the original fastening. Therefore, regular strap adjustment during daily movements appears to be helpful and essential to prevent the biomechanical action of spinal orthosis on the trunk from becoming less effective. Interestingly, Lou et al. have developed a prototype intelligent orthosis with the strap tension being controlled and maintained at the optimal prescribed level [39]. However, this technique has not been applied to real cases because of the limitation on the design of the motorized system. In addition, the creeping effect of the materials of the strap might also influence the strap tension during spinal orthosis wearing and daily activities, but there is no study in this area yet, which could be one of the future research directions.

Mac-Thiong and his colleagues [7] claimed that for right thoracic curves, the strap tension should be set high enough, to a maximum of 60 N, and the strap tension should be about 40 N for right thoracic with left lumbar curves. However, the strap tightness should not be at the cost of the patient’s comfort and compliance with the spinal orthosis. Too loose straps are ineffective, but too tight straps could possibly cause health issues [40]. A notable increase in sternal pressure may be suffered as the strap tension increases, which may restrict chest expansion, impede breast development, and deteriorate pulmonary function [41,42]. Excessive abdominal pressure should also be aware as it may be linked to reflux esophagitis [43,44] and renal dysfunction [45]. Therefore, balancing the strap tension with the patient’s comfort is crucial for ensuring both effectiveness and patient compliance. According to Li et al.’s findings, the patient-specific 3D-printed orthosis was more comfortable than the conventional ones [46]; this customization enhanced comfort while maintaining the necessary corrective effect. Moreover, gradually increasing strap tension allows patients to adapt to the orthosis, reducing initial discomfort and enabling them to tolerate higher tensions over time. Conducting regular radiographic examinations and clinical assessments to monitor the corrective outcomes and patient comfort could also promote the strap tension adjustments [47]. Additionally, clear communications with patients and their families about the importance of orthosis wearing, including the expected discomfort and treatment outcomes, and providing psychosocial support for patients may improve patient’s treatment experience and increase their tolerance [48].

### 4.2. Pad Pressure

Additionally, this study analyzed the interface pressure acting through the pads of the orthosis upon the body. The thoracic pad had a median value of 8.92 kPa (IQR: 7.99 to 9.34), and the thoracolumbar/lumbar pad pressure had a median of 7.51 kPa (IQR: 7.04 to 41.2). Pressure pads are used in orthosis to modify the location and intensity of the mechanical forces supporting the torso and correcting the spinal deformity. The direct orthosis–trunk compressive forces provide an optimal fit for the individual orthosis and rectify the distortion. Yang et al. [49] stated that the pressure pads provided the majority of the external correction force when the orthosis was worn tightly, and they designed an airbag system for replacing the pads and providing correction force. An approximate 9 kPa of interface pressure was measured and significant correction in the Cobb angle was observed during the follow-up [49]. This pressure was consistent with the findings of Wong’s study (70 mmHg, approximately 9 kPa) [32]. Similarly, Chase et al. measured the interface pressure at the compression pads within the orthosis and identified an approximate value of 8kPa with an initial curve correction rate of 37% [20]. However, the pressures measured in Ahmad et al.’s study were 82.5 kPa and 85.1 kPa for the thoracic and thoracolumbar/lumbar pads, respectively [34]. The acquisition of these values might be attributed to the design of the orthosis based on the principle of overcorrection and the young age of the patients who have better spinal flexibility to tolerate the high pressure. Furthermore, the results were derived under the condition of maximum strap tension, leading to pressure values that were considerably higher than those observed in other studies. This review analyzed the median and IQR of the pad pressure rather than the mean value, mainly due to the substantial values extracted in Ahmad’s study [34], which introduced extreme values into the dataset of the studies included within this review. Relative to the mean, the use of the median and IQR is more robust against the influence of outliers, thereby providing a more representative outcome. Moreover, this review identified the big range of interface pressure at the thoracic region and thoracolumbar/lumbar region, which might be explained by the different curve patterns, the orthosis types, spinal flexibilities, and other potential factors.

Curve patterns could affect the interface forces acting on the body. The included studies had different descriptions of the curve patterns. Chase et al. and Mulcahy et al. reported the curve patterns as single curves or double curves [20,37], while the other studies reported the direction and the region of the curves [7,26,32,33,34,36]. A more detailed description of the curve patterns would be better for the interpretation of the corrective pressure and its effect. Pham et al. [26] found that the overall average pressure for patients with right thoracic curves was 7.99 kPa, while for patients with right thoracolumbar curves, it was 7.04 kPa. Loukos et al. and other studies also reported different interface force values among different curve patterns [7,27,36,37]. Furthermore, Chou and his team demonstrated that both lumbar and thoracic scoliosis curves were more effectively reduced with a high thoracic pad [50]. These findings were not difficult to understand since the biomechanical principle for scoliosis correction was based on the curve patterns. This review found that the thoracic pad pressure of the single curve (8.55 kPa) was greater than that of the double curves (7.06 kPa), but the thoracolumbar/lumbar pad pressure was approximate for the single curve (7.11 kPa) and double curves (7.84 kPa). A single curve may require more concentrated pressure for correction compared to double curves, especially for a single thoracic curve, which can be addressed with a more distributed pressure system. In patients with a single curve, sufficient force must be applied by the orthosis pad to correct the primary curve, whereas in double curves, the forces are applied to correct both the thoracic and lumbar curves, potentially resulting in a more balanced pressure distribution [51]. Still, the current research lacks a comprehensive exploration of how different curve types affect the pressure and corrective outcomes of orthoses. Future studies could benefit from focusing more on this area to enhance our understanding of the biomechanical interactions between the orthosis and the spine.

Different orthosis types could produce different corrective forces. The studies included in this review contained two Chêneau orthoses [26,34], four Boston orthoses [7,20,27,35], and two Milwaukee orthoses [32,37]. When excluding the outliers from the two studies [27,34], it is observed that the Milwaukee orthosis demonstrated a higher pressure at the thoracic pad (9.08 kPa) in comparison to the Chêneau orthosis (7.99 kPa), while the Chêneau orthosis showed greater pressure than the Boston orthosis (7.22 kPa). This hierarchy of pressure distribution could potentially be correlated with the design principle inherent to each orthosis. The Milwaukee orthosis exerts axial compression along the spine and applies pressure to specific points on the body, which mainly provides passive external force to the body [18,52], thus creating more pressure through the pad, while the Chêneau orthosis intends to actively control the curve progression by reminding the patients to shift away from pressure areas, asymmetrically guiding respiratory movements, the repositioning of the spatial arrangement of trunk muscles, and the anti-gravitational effect [53]. Boston orthosis is usually designed with a low profile and symmetrical style, allowing freedom of movement away from the corrective forces [54]. However, there are few comparative studies related to the corrective mechanisms and force magnitudes of different types of orthoses, and future research should explore more in this field.

Spinal flexibility refers to the degree to which a spinal abnormality can be corrected and intrinsically signifies the spine’s capacity to adapt to external forces [55]. As an important influential factor in corrective outcomes of spinal orthosis [56], spinal flexibility could also impact the magnitudes of corrective forces [57,58]. Nevertheless, the included studies rarely reported the spinal flexibility of the patients, and this should be discussed more deeply in future studies.

In addition, positions and daily activities could also influence the change in the interface forces [22,32,33,36,38,59]. Physical exercise during orthosis wearing was also proven to produce a better biomechanical action on spinal curve correction [24]. To obtain a better corrective outcome from spinal orthosis, these factors should be considered as well. Furthermore, Wynarsky and Schultz showed that the corrective effect produced by the orthosis was based on the position, strength, and direction of the forces applied to the spine [18]. Therefore, more attention should be paid to the location and direction of the corrective forces as well.

### 4.3. Corrective Outcomes

In this review, nine articles reported the corrective outcomes under the reported biomechanical situations. The correction rates ranged from 14% [35] to 44% [26]. However, some studies reported 6-month out-of-orthosis correction rates, and some correction rates were more than one year. Additionally, Chase et al. [20] and Pham et al. [26] observed an approximate interface pressure (around 8 kPa), but showed different correction rates of 15% and 44% at the 6-month follow-up session. The reason for such a considerable correction might be the different kinds of orthosis used in the two studies. Meanwhile, Loukos et al. displayed that there was no significant correlation between the mean pressure in the standing position and the amount of curve correction for both thoracic curves and thoracolumbar curves [36]. Van den Hout and his colleagues (2002) also concluded that the magnitude of the pad force was not significantly correlated with the correction outcomes [27]. These findings were contradictory with the conventional beliefs that greater controlling force can produce more curve correction within a certain range, and further research is merited to confirm this issue.

In addition to the controlling force, it is well accepted that the corrective outcomes of spinal orthosis could be correlated with many other factors, such as curve magnitude [60], skeletal maturity [60], orthosis wearing compliance [3,61], and curve flexibility [56]. Therefore, unraveling the major factors that contribute to better correction is important and could ultimately result in a more individualized orthotic treatment.

### 4.4. Limitations

Some limitations should be considered when understanding the results. First, the scoping of this review was confined to the English-language literature, which may have constrained the number of studies eligible for inclusion, thereby potentially introducing bias into the research findings. In addition, it was hard to derive an exact value of strap tension and pad pressure as a reference for future studies because of the heterogeneity among the studies, including variations in orthosis types, measurement methods, pad types (T or TL/L pads), and study samples. Additionally, these studies rarely reported the correlation between the controlling force magnitudes and the correction outcomes, resulting in the failure to determine how much controlling force could yield better corrective results in this review.

## 5. Conclusions

This review comprehensively analyzed 10 relevant studies and identified the range for strap tensions of spinal orthosis to be from 26.8 N to 60.4 N, with a mean value of 42.5 N. For patients aged 12 to 13 with moderate curves (Cobb angle between 25 and 40), the initial strap tension is recommended to be around 26.8 N, while for patients aged 13 to 14, 48.2 N is advised. The interface pressure from the thoracic pads had a median of 8.75 kPa (IQR: 7.53–9.16), and the median pressure magnitude of the thoracolumbar and lumbar regions was 7.11 kPa, with an IQR of 5.92 to 7.51 kPa. For patients aged 14–15 years, or those with double curves, it is recommended that the thoracic pad pressure be maintained at approximately 7.06 kPa, while the lumbar pad pressure could be set at 7.84 kPa. For patients aged 13–14 years, the thoracic pad pressure is suggested to be set at 7.89 kPa, with the lumbar pad pressure considered at 6.34 kPa. In contrast, for children with scoliosis aged 12–13 years, the thoracic pad pressure is recommended to be around 9.09 kPa. The findings suggest that tighter straps generally result in higher pressure on the body, potentially leading to more effective corrective outcomes. Orthotists can refer to these findings to optimize orthosis design by calibrating strap tensions to provide adequate controlling forces while balancing patient comfort and compliance.

## Figures and Tables

**Table 1:** Characteristics of included studies.

Reference	Country/Region	Study Setting	Sample Size	Gender	Age (Y)	Curve Pattern	Initial Cobb (°)	Correction Rate
Wong 1998 [32]	Hong Kong	Lab	9	9 F	13.3	9D	36	36% (>3 m)
Wong 2000 [33]	Hong Kong	Lab	26	26 F	12.9	15RT/1LT/8LL/2LTL	34.4	31% (20 m)
Ahmad 2019 [34]	Malaysia	Clinic	72	60 F/12 M	11.4	72D	30.5	29% (6 m)
Chase 1989 [20]	England	Clinic	14	12 F/2 M	14.3	5D/9S	37.6	15% (6 m)
Jiang 1993 [35]	Canada	Clinic	8	8 F	9–14	8S	29	14% (21 m)
Loukos 2011 [36]	Greece	Clinic	44	38 F/6 M	13.8	44S	27.8	30% (6 m)
Mac-thiong 2004 [7]	Canada	Clinic	41	37 F/4 M	13.5	27D/14S	35.6	NA
Mulcahy 1973 [37]	The USA	Clinic	30	26 F/4 M	12.7	12D/18S	38.8	22% (17 m)
Pham 2008 [26]	France	Lab	32	30 F/2 M	13.5	18RT/14RTL	32.6	44% (6 m)
Van den Hout 2002 [27]	The Netherlands	Clinic	16	14 F/2 M	13.7	16D	31	43% (6.5 m)

F: female; M: male; Y: year; RT: right thoracic; LT: left thoracic; LL: left lumbar; LTL: left thoracolumbar; RTL: right thoracolumbar; D: double curve; S: single curve; m: month; NA: not available.

**Table 2:** MINORS results of the included studies.

Study/Items	A Clearly Stated Aim	Inclusion of Consecutive Patients	Prospective Collection of Data	Endpoints Appropriate to the Aim of the Study	Unbiased Assessment of the Study Endpoint	Follow-Up Period Appropriate to the Aim of the Study	Loss to Follow Up Less than 5%	Prospective Calculation of the Study Size	Overall Scores
Wong 1998 [32]	2	2	2	2	2	2	2	0	14
Wong 2000 [33]	2	2	2	2	2	2	1	0	13
Ahmad 2019 [34]	2	2	2	2	2	2	2	0	14
Chase 1989 [20]	2	2	2	2	2	2	1	0	13
Jiang 1993 [35]	2	2	2	2	2	2	2	0	14
Loukos 2011 [36]	2	2	2	2	2	2	0	0	12
Mac-thiong 2004 [7]	2	2	2	2	2	2	2	0	14
Mulcahy 1973 [37]	0	2	2	0	2	2	2	0	10
Pham 2008 [26]	2	2	2	2	2	2	0	0	12
Van den Hout 2002 [27]	2	2	2	2	2	2	2	0	14

The items are scored 0 (not reported), 1 (reported but inadequate), or 2 (reported and adequate). The overall ideal score is 16 for non-comparative studies.

**Table 3:** Summary of the strength of strap tension.

Reference	Strap	Orthosis Type	Tension (N)	Measurement Devices
Wong 1998 [32]	Thoracic strap	Milwaukee	36 ± 11	Purpose-designed buckle force-transducers
Wong 2000 [33]	Axillary/thoracic/lumbar/pelvic strap	Hong Kong orthosis	26.8 ± 5.2	Purpose-designed buckle force-transducers
Jiang 1993 [35]	All straps	Boston	37 ± 22	Force transducer with a strain gauge
Mac-Thiong 2004 [7]	Thoracic strap	Boston	60.4 ± 5.8	A tension-load cell with a data logger

N: Newton.

**Table 4:** Summary of the thoracic and thoracolumbar/lumbar pad pressure.

Author (Year)	Orthosis Type	Pressure (kPa)-T	Pressure (kPa)-TL/L	Measurement Devices
Wong 1998 [32]	Milwaukee	9.31	NA	Electrohydraulic sensors—DPM
Wong 2000 [33]	Hong Kong orthosis	9.34	NA	Electrohydraulic sensors—the Dynamic Pressure Monitor(DPM)
Ahmad 2019 [34]	Cheneau	82.5	85.1	F-Socket transducers (9811E) and Tekscan system
Chase 1989 [20]	Boston	7.06	7.84	Qxford pressure monitor
Jiang 1993 [35]	Boston	9.00	NA	Qxford pressure monitor
Loukos 2011 [36]	DDB	8.66	7.17	F-Socket 9801 pressure sensor
Mac-Thiong 2004 [7]	Boston	5.60	4.80	Flexible pressure mat made of 192 force-sensing transducers
Mulcahy 1973 [37]	Milwaukee	8.84	NA	Force-sensing element
Pham 2008 [26]	Cheneau	7.99	7.04	TekScan system
Van den Hout 2002 [27]	Boston	15.7	41.2	The electronic Pedar measuring device

TLSO: thoraco-lumbo-sacral orthosis; T: thoracic; TL/L: thoracolumbar/lumbar; DDB: dynamic derotation brace; NA: not available.

**Table 5:** Summary of the pad pressure and strap tensions of different factors.

Factors	Pressure (kPa)-T	Pressure (kPa)-TL/L	Strap Tension (N)
Orthosis type			
Milwaukee orthosis	9.08	NA	36
Boston orthosis	9.34 ^a^/7.22 ^b^	17.95 ^a^/6.32 ^b^	48.7
Chêneau orthosis	45.25 ^a^/7.99 ^b^	46.07 ^a^/7.04 ^b^	NA
Hong Kong orthosis	9.34	NA	26.8
DDB orthosis	8.66	7.17	NA
Age group (Y)			
11–12	82.5 ^a^	85.1 ^a^	NA
12–13	9.09	NA	26.8
13–14	9.45 ^a^/7.89 ^b^	15.05 ^a^/6.34 ^b^	48.2
14–15	7.06	7.84	NA
Curve patterns			
Single curve	8.55	7.11	37
Double curves	35.09 ^a^/7.06 ^b^	44.71 ^a^/7.84 ^b^	36

^a^: with outlier; ^b^: without outlier; Y: year; NA: not available.

## Data Availability

The original contributions presented in this study are included in the article. Further inquiries can be directed to the corresponding author.
